# 

**Published:** 1919-04

**Authors:** 


					﻿VOL. XXXIV.
APRIL, 1919.
No. 10.
Texas
Medical
Journal
Entered at the postoffice at Austin, Texas, as second-class mail matter
				

